# Assessment of endothelial colony forming cells delivery routes in a murine model of critical limb threatening ischemia using an optimized cell tracking approach

**DOI:** 10.1186/s13287-022-02943-8

**Published:** 2022-06-21

**Authors:** Marta Rojas-Torres, Ismael Sánchez-Gomar, Antonio Rosal-Vela, Lucía Beltrán-Camacho, Sara Eslava-Alcón, José Ángel Alonso-Piñeiro, Javier Martín-Ramírez, Rafael Moreno-Luna, Mª Carmen Durán-Ruiz

**Affiliations:** 1 Biomedicine, Biotechnology and Public Health Department, Cádiz University, Cádiz, Spain; 2 Institute of Research and Innovation in Biomedical Sciences of Cádiz (INiBICA), Cádiz, Spain; 3 Preclinical R&D Department at Ixaka Iberia SL, Seville, Spain; 4 Laboratory of Neuroinflammation, Hospital Nacional de Paraplejicos, SESCAM, Toledo, Spain

**Keywords:** Biodistribution, CLTI, Cell therapy, ECFCs, DiR labeling

## Abstract

**Background:**

Endothelial colony forming cells (ECFCs), alone or in combination with mesenchymal stem cells, have been selected as potential therapeutic candidates for critical limb-threatening ischemia (CLTI), mainly for those patients considered as “no-option,” due to their capability to enhance revascularization and perfusion recovery of ischemic tissues. Nevertheless, prior to translating cell therapy to the clinic, biodistribution assays are required by regulatory guidelines to ensure biosafety as well as to discard undesired systemic translocations. Different approaches, from imaging technologies to qPCR-based methods, are currently applied.

**Methods:**

In the current study, we have optimized a cell-tracking assay based on DiR fluorescent cell labeling and near-infrared detection for in vivo and ex vivo assays. Briefly, an improved protocol for DiR staining was set up, by incubation of ECFCs with 6.67 µM DiR and intensive washing steps prior cell administration. The minimal signal detected for the residual DiR, remaining after these washes, was considered as a baseline signal to estimate cell amounts correlated to the DiR intensity values registered in vivo. Besides, several assays were also performed to determine any potential effect of DiR over ECFCs functionality. Furthermore, the optimized protocol was applied in combination with qPCR amplification of specific human Alu sequences to assess the final distribution of ECFCs after intramuscular or intravenous administration to a murine model of CLTI.

**Results:**

The optimized DiR labeling protocol indicated that ECFCs administered intramuscularly remained mainly within the hind limb muscle while cells injected intravenously were found in the spleen, liver and lungs.

**Conclusion:**

Overall, the combination of DiR labeling and qPCR analysis in biodistribution assays constitutes a highly sensitive approach to systemically track cells in vivo. Thereby, human ECFCs administered intramuscularly to CLTI mice remained locally within the ischemic tissues, while intravenously injected cells were found in several organs. Our data corroborate the need to perform biodistribution assays in order to define specific parameters such as the optimal delivery route for ECFCs before their application into the clinic.

**Supplementary Information:**

The online version contains supplementary material available at 10.1186/s13287-022-02943-8.

## Background

Critical limb-threatening ischemia (CLTI) constitutes a debilitating disease caused by the narrowing and obstruction of the primary systemic arteries, mainly due to atherosclerosis. CLTI patients suffer from chronic rest pain, ischemic ulcers, gangrene and, eventually, toes or extremities amputation, significantly impairing their quality of life [1, 2]. The prevalence and incidence of this disease is constantly growing, representing a great burden for public health [3]. Despite significant advances in surgical treatments (i.e., bypass or angioplasties) [4], the percentage of patients that could undergo surgical revascularization is low, principally due to related comorbidities [5, 6]. In addition, some patients require re-intervention and, in the last term, they might need amputation. Alternatively, angiogenic cell therapy has emerged as a feasible option for ischemic diseases because of its potential to enhance revascularization and blood flow recovery, returning oxygen and nutrients towards the affected areas [7–9]. Different strategies, including the administration of endothelial colony-forming cells (ECFCs), are currently under investigation, mainly with “no-option” CLTI patients [10]. ECFCs have the ability to augment collateral revascularization and reperfusion [11, 12], representing a suitable candidate to treat ischemic diseases such as CLTI.

Before these and other cell treatments can be translated into the clinic, several preclinical assays are required by regulatory authorities to ensure the safety and efficacy of cell-based medicinal products [13, 14]. These therapies should guarantee the highest cell availability for therapeutic effects within the target tissue, as well as the avoidance of possible undesirable effects as result of the translocation to other parts of the body. Therefore, cell tracking assays are required to decipher the systemic biodistribution of cells, which might depend, among others, on the injection route. Likewise, other factors such as cell size or source, immunological features and labeling, detection method or the animal model could also affect the pharmacokinetics of cells [15].

Currently, multiple cell-tracking strategies are applied. Amid all, cell transfection with fluorescent proteins allows visualization via microscopy and provides quantitative information regarding cell localization [13]. However, the transfection efficacy might affect cell expression and further follow-up at the long term [16, 17]. Alternatively, the use of nanoparticles for cell labeling does not compromise intrinsic cell processes and offer adequate spatial resolution, although these methods require specific equipment and trained personnel, not always applicable to all facilities [18, 19]. Instead, optical imaging is widely used in preclinical models, providing excellent sensitivity without cell compromising or without high-tech equipment requirements. In this regard, fluorescence and bioluminescence are the two major methods applied 20–23]. The near-infrared (NIR) dye carbocyanine 1,1-dioctadecyl-3,3,3,3 tetramethylindotricarbocyanine iodide (DiR) has been commonly used with a variety of cells, including mesenchymal stem cells (MSCs) [24, 25], macrophages [26, 27], cardiospheres [28], extracellular vesicles [29] or epithelial cells [30]. This lipophilic NIR fluorescent dye gets inserted into cell membranes resulting in a specific and stable signal as a result of high cellular uptake. Moreover, NIR properties are beneficial for in vivo cell imaging and tracking, due to reduced animal auto-fluorescence at higher wavelength [31, 32]. Its lipophilic condition makes cell labeling requirements equivalent to physiological environment, not damaging cell viability. Despite all this, a thorough optimization of the labeling protocol is required, as any residual dye remaining in the injection solution could stain other tissues and provide false positive fluorescent signals.

Herein, we have performed an optimized DiR labeling protocol for ECFCs, to avoid any false positive signals during preclinical biodistribution assays. Furthermore, this strategy was applied to compare two alternative biodistribution routes for ECFCs, intramuscular (IM) and intravenous (IV) administration, in a murine model of CLTI, evaluating, simultaneously, two complementary strategies for cell tracking: DiR labeling and qPCR amplification of specific Alu sequences, as previously described [33–35].

## Methods

### ECFCs isolation, culture and characterization

ECFCs were isolated from human umbilical cord blood as described [36–38] and cultured in 1% gelatin-coated plates using ECFCs-medium: EGM-2 (except for hydrocortisone; Lonza) supplemented with 20% FBS, 1X Penicillin/Streptomycin (P/S). ECFCs between passages 5 and 7 were used for all experiments.

Cell identity was confirmed by testing cloning-forming ability, as described [39]. Also, several markers were analyzed by flow cytometry with the Cytoflex (Beckman Coulter) cytometer and CytoExpert software: CD14, CD31, CD34, CD45, CD73, CD90, CD133, CD146 and CD309. An isotype IgG1 antibody was used as a negative control. Data were analyzed with FlowJo v10.4 software. The full list of antibodies can be found in supplementary materials (Additional file 1: table S1), as well as the results of ECFCs characterization by flow cytometry (Additional file 1: figure S1).

### Animals

Balb-C Nude (CAnN.Cg-Foxn1nu/Crl) mice (n:16), age 9–12 weeks, male and female (in equal numbers), were produced and maintained under standard laboratory conditions at the Animal Core Facility (SEPA), Cadiz University. Mice were allocated in special rooms, with technical staff constantly supervising filters and air recirculation and monitoring any signs of ill-health. Animals were fed sterile standard chow diet ad libitum and had free access to sterile water. No animal was sacrificed prematurely during the experiments.

### Optimization of DiR labeling

#### Determination of the relation between DiR intensities and cell numbers

First, a standard curve was designed to determine the number of cells based on DiR intensity values. Thus, 1·10^6^ ECFCs were labeled with 6.67 µM DiR (Biotium, #60,017) in 1 ml of PBS 1X during 25 min at 37 °C, following standard guidelines. After that, three consecutive washing steps were performed with 1 ml of PBS 1X, including centrifugation (5 min, 500 g), discarding supernatants and washing cells. Next, different amounts of ECFCs (1/2 serial dilutions) were seeded in a 12-well plate: 200,000, 100,000, 50,000, 25,000, 12,500, 6250, 3125, 1562 and 781 cells. The plate was scanned in the Odyssey image NIR acquisition system (Licor Biosciences). Parameters used for the scan: Intensity 2 and detectors in 700 nm and 800 nm activated. Intensity values were registered on day 1, day 2 and day 3 after seeding as K counts/mm^2^, and standard curves were obtained per day (in triplicates) with excel.

#### Determination of the optimal DiR staining protocol

**Fig. 1:**
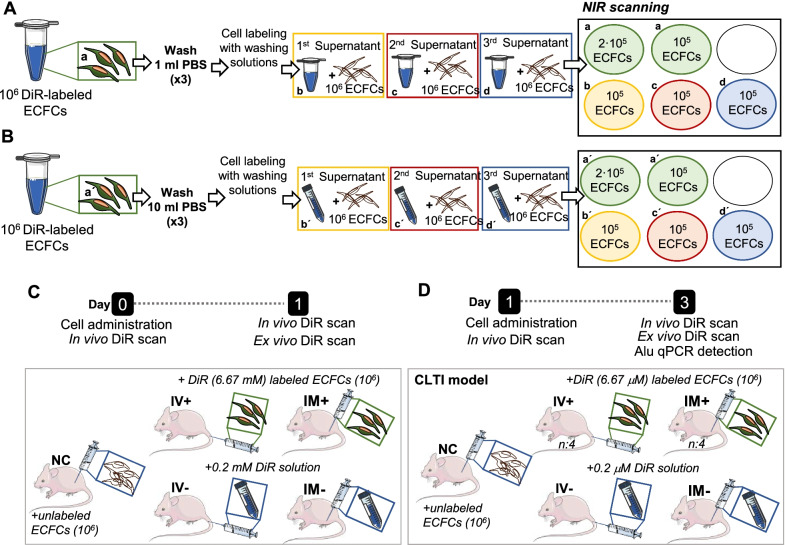
Schematic representation of assays work-flow. Optimization of DiR labeling steps. **A** ECFCs (1·10^6^) were labeled with 6.67 µM DiR, and 2·10^5^ and 1·10^5^ cells (a) were plated after 3 consecutive washes with 1 ml PBS. Also, 3 sets of ECFCs (1·10^5^ each) were labeled with the consecutive supernatant solutions from washing steps (b-d). **B** DiR- labeled ECFCs (1·10^6^) were washed three times with 10 ml PBS 1X, and 2·10^5^ and 10^5^ cells were plated (a´). Again, sets of 10^5^ ECFCs were labeled with the supernatant solutions and seeded (b´- d´). **C** Balb-c nude mice were injected with DiR-labeled ECFCs (1·10^6^ in 50 µl), intravenous (IV +) or intramuscularly (IM +), or with 50 µl of the third washing supernatant solution (10 ml PBS each) applied to these cells (IV- and IM-). In vivo scans were performed on day 0 and 1. An ex vivo scan was carried out on day 1. **D)** CLTI Balb-c nude received 10^6^ DiR-labeled ECFCs (50 µl) intravenously (IV + , n:4) or intramuscularly (IM + , n:4). Also, a DiR residual negative control was included, consisting in CLTI mice receiving via tail vein (IV-, n:1) or intramuscularly (IM-, n:1), 50 µl of 0.2 µM DiR, equivalent to the third supernatant washing solution. An additional mouse was transplanted with 10^6^ unlabeled ECFCs to consider background signal (NC)

We next evaluated whether the solution remaining from the three washing steps after DiR labeling could still stain other cells. In total, 1·10^6^ ECFCs were labeled with 6.67 µM DiR in 1 ml of PBS 1X, 25 min, 37 °C. Cells were centrifuged (5 min, 500 g), the supernatant was collected in a new tube, and cells were washed with 1 ml of PBS 1X. This washing step was repeated twice, collecting also the supernatants from each wash. Then, 2·10^5^ and 1·10^5^ ECFCs from the initial DiR labeling (and after three consecutive washes) were seeded in a 12-well plate. Additionally, other sets of ECFCs (1·10^5^) were incubated each one with the solutions collected from the three consecutive washing steps, 25 min at 37 °C, plated and scanned as previously indicated to determine residual staining (Fig. 1A).

Next, we proceeded as before, incubating 1·10^6^ cells with DiR 6.67 µM in 1 ml of PBS 1X, 25 min at 37 °C, but washing three times with 10 ml of PBS 1X. Again, supernatants from each washing step were collected and ECFCs (1·10^5^) were incubated with them, to determine if the remaining DiR solution could still preserve the capacity to stain cells (Fig. 1B).

#### Determination of DiR residual concentrations

In order to calculate the concentration of DiR equivalent to the one observed in cells pre-labeled with the third wash step (residual DiR), a standard curve was built by incubating ECFCs (1·10^5^), 25 min and 37 °C, with serial DiR dilutions (1/2), ranging from 3.34 µM up to 0.0016 µM. Cells were scanned as described above, together with a negative control, consisting of 1·10^5^ unlabeled cells, to consider background levels.

#### Determination of DiR residual staining concentrations in vivo and ex vivo

Afterward, we tested whether the DiR residual intensity detected in vitro could also have any signal in vivo (Fig. 1C). Again, 1·10^6^ ECFCs were labeled with 6.67 µM DiR, 25 min at 37 °C, followed by three washing steps with 10 ml PBS 1X. After centrifugation, the supernatant from the third wash (DiR residual) was collected. Balb-c nude mice (n:5) were anesthetized administering intraperitoneally xylazine (10 mg/kg) and ketamine (100 mg/kg), and 1·10^6^ DiR-labeled ECFCs (resuspended in 50 µl sterile saline serum) were injected intravenously (via tail, IV +) or intramuscularly (left hind limb, IM +) as positive controls. In addition, another set of mice were injected, intravenous (IV-) and intramuscularly (IM-), with 0.2 µM of DiR, equivalent to the third wash (DiR residual). Finally, a negative control with 1·10^6^ unlabeled ECFCs was used to consider background signals (NC).

On day 0 and day 1 after cell administration, mice were anesthetized and transferred to the NiR scanning system (Odyssey, LI-COR Biosciences), and in vivo images were taken, registering the DiR intensities from the areas of thorax, abdomen, both limbs and paws. Mice were sacrificed in a CO_2_ chamber (day 1), and several tissues and organs were extracted: left and right limbs, spleen, kidneys, liver and lungs. They were all immediately scanned with the parameters indicated above.

DiR fluorescence values from in vivo and ex vivo assays were calculated per organ/area of scanned regions. Experimental values were normalized *versus* the NC, which was taken as baseline (0 intensity) and after that fluorescence intensity values of IV- and IM- (residual DiR negative controls) were subtracted from IV + and IM + intensities. Fluorescent intensities were registered as counts/mm^2^. Full information regarding fluorescence intensities in vivo and ex vivo can be found in Additional file 1: Tables S2-S3.

### In vitro analysis and biocompatibility

#### Proliferation assay

DiR-labeled (6.67 µM DiR) and unlabeled 1·10^5^ ECFCs (n:4) were plated in 12-well plates pre-coated with 1% gelatin, and cultured in ECFCs-medium for 48 h, as described above. Next, cells were fixed in 4% paraformaldehyde (PFA), permeabilized with 0.2% triton and blocked with 5% BSA and 0.1% triton, followed by incubation with the primary antibody anti-α-Ki67 (1:500, PA5-16,785, Invitrogen), at 4 °C overnight, and then with the specific secondary antibody (1:500, A-21428, ThermoFisher), 1 h, at room temperature in the dark. Finally, nuclei were stained with DAPi (0.2 µg/ml). Images were acquired from random sections (n:5) of each well, using MMI CellCut Plus (Olympus), at 20X magnification, and then analyzed with the Zen 2 (Zeiss) software. The proliferation assay as well as the other in vitro assays was performed with ECFCs from one single donor, repeated four times. The results were expressed as the percentage of total ECFCs that were positive for Ki67.

#### Angiogenesis assay

Briefly, 1·10^4^ DiR-labeled or untreated ECFCs (n:4) were seeded, in triplicates, into a µ-plate angiogenesis 96 well (ibidi, 89,646) pre-coated with 10 µl Matrigel (BD Bioscience, 256,231), as described [39]. Images were registered after 24 h, 48 h and 72 h, with a Moticam 3.0 camera connected to an inverted phase-contrast microscope, under 4X magnification. The number of meshes and total length of segments in each well were quantified using the Angiogenesis Analyzer plugin with Image J v.2.0.

### Apoptosis assay

ECFCs (1.5·10^5^) were labeled with 6.67 µM DiR (n:4) or left unlabeled (n:4) and then seeded in triplicates in 12-well plates, and cultured 48 h at 37ºC, 5% CO_2_. Cells were detached with trypsin and incubated with 5 µl propidium iodide (PI, #556,463, BD) and 5 µl Annexin-V (AV, PB-V450, #56,056, BD) at 4 °C, 30 min, as described [39]. Apoptotic cells were analyzed by flow cytometry as indicated above.

#### Co-culture assay

ECFCs (1·10^5^) were labeled with 6.67 µM DiR, washed with 10 ml PBS 1x, as described above, seeded in a 1% gelatin-coated 12-well plate and then incubated for 24 h, 37 °C, 5% CO_2_ in ECFCs-medium. In parallel, Jurkat cells were grown in RPMI medium supplemented with 10% FBS, 1X P/S, 20% glutamine, collected and washed twice with PBS 1X, and then plated with the pre-labeled ECFCs, co-culturing Jurkat and DiR ECFCs labeled cells in the aforementioned ECFCs conditions. After 24 h, Jurkat cells were collected and labeled with PE- α-human CD3 antibody (Biolegend, #317,308), 25 min at 4 ºC. Also, ECFCs were collected through trypsinization and labeled with α-human CD31 antibody (Biolegend, #303,103). Fluorescence was measured by flow cytometry, as described above. The mean fluorescence intensities were registered and the percentage of DiR + cells was calculated.

### Biodistribution assays of ECFCs in CLTI mice

#### Murine model of CLTI and administration of DiR pre-labeled ECFCs

Balb-c nude mice (n:11) were anesthetized with xylazine (10 mg/kg) and ketamine (100 mg/kg), administered intraperitoneally before surgery, and double ligation was performed occluding the distal and proximal ends of the left femoral artery with double knots of suture (non-absorbable 6/0), as previously described [33, 34, 40]. Mice received ketoprofen (2 mg/kg) intraperitoneally as analgesic for three consecutive days.

Twenty-four hours after surgery, eight sets of 1·10^6^ ECFCs were pre-labeled as indicated before (6.67 µM DiR), washing three times with 10 ml PBS. Then, CLTI mice were injected with 1·10^6^ DiR-labeled cells (in 50 µl sterile saline serum) either intravenously (via tail, IV + , n:4) or intramuscularly (IM + , n:4), with 3–4 injections in different points (back and front) around the femoral artery. Also, another 2 mice were injected with 0.2 µM of residual DiR as negative control (IV-, IM-), together with a NC mouse, with unlabeled cells (Fig. 1D).

#### Near-infrared scanning and data analysis

In vivo scans were performed on day 1 and day 3 as described before, registering the DiR signal intensities from the areas of thorax, abdomen, as well as both limbs and paws. On day 3, after the in vivo scanning, mice were sacrificed in a CO_2_ chamber, and several tissues and organs were extracted and scanned, as indicated above. DiR fluorescence values from in vivo and ex vivo assays were calculated as described in the previous section “Determination of DiR residual staining concentrations in vivo and ex vivo”.

Following the scanning, muscles from the back (gastrocnemius and sole muscle) and front side (tibialis) of the left and right limb surrounding the femoral artery were extracted and snap-frozen in liquid nitrogen for further qPCR analysis of human-specific Alu (hAlu) sequences or fixed in 4% PFA for further immunohistochemistry (IHC).

#### qPCR-Alu sequence analysis

The presence of human cells (hcells) within the tissues was also determined by quantification of specific hAlu sequences, as previously described [33–35]. Briefly, snap-frozen tissues were homogenized before genomic DNA isolation from tissue samples (hind limbs, kidney, liver, lungs and spleen) using E.Z.N.A Tissue DNA Kit (OMEGA bio-tek).

Linearity and resolution limits were determined by mixing human genomic DNA with murine DNA at several concentrations of human DNA (hDNA) in 100 ng of total genomic DNA. The sensitivity of the assay allowed to detect 1 human cell in 10,000 mouse cells. Alu-qPCR was performed, in triplicates, using TaqMan Universal Master Mix II, no UNG (Thermo Fisher Scientific), 0.2 μM forward and reverse primers and 0.25 μM hydrolysis probe on a CFX Connect Real-Time System (Bio-Rad), as described [33–35].Data were analyzed with CFX Manager 3.1 (Bio-rad).

The total number of ECFCs in the different organs was estimated as previously described [33–35]. Values obtained for hDNA in 100 ng were extrapolated to total hDNA extracted per mg of tissue and considering the relation of 5 pg of DNA per hcells, as described [41].

### Detection of DiR-labeled cells by immunohistochemistry

ECFCs distribution among the ischemic tissues was also evaluated by IHC. Thus, frontal tissues from left limbs extracted three days after cell administration were fixed with 4% PFA for 72 h and dehydrated before OCT embedding. Tissues were then transversally sectioned (8 µm) and IHC was performed to detect endothelial human cells and blood vessels with specific antibodies against CD31 and α-smooth muscle actin (α-SMA), respectively, as described [33, 34]. Full information regarding the antibodies employed can be found in Additional file 1: Table S1. Images were taken at 40 × using a Zeiss LSM 880 confocal microscope. Images were analyzed and processed with the Zen 2 (Zeiss) software.

### Statistical analysis

Statistical analysis was performed using GraphPad Prism v.9 software. Data were verified for normal distribution using Shapiro–Wilk normality test. Differences between groups were calculated with Kruskal–Wallis test and Dunn’s test as post hoc analysis. Data were represented as the mean ± SEM, indicating the individual points for each data set. Differences were considered as statistically significant with *p*-values < 0.05. Full information including *p*-values from each data set analysis is shown in Additional file 1: Tables S4-S12.

## Results

### Optimization of the DiR labeling protocol

#### Relation between DiR intensities and cell numbers

**Fig. 2:**
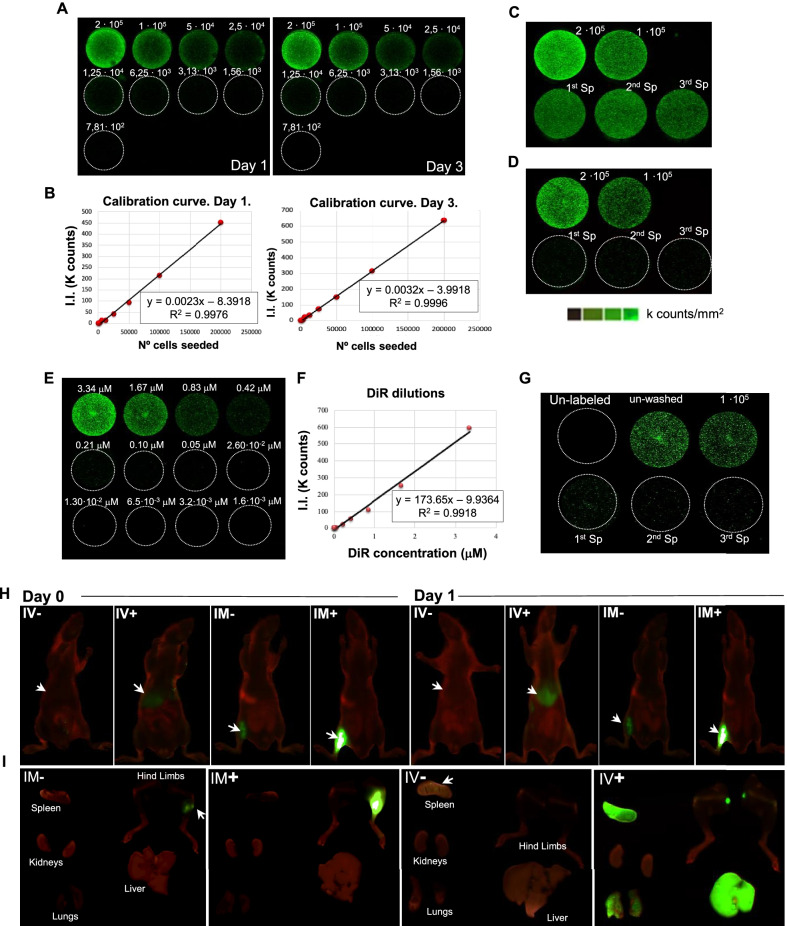
Determination of DiR residual staining. **A** Representative scans from calibration curve on days 1 and 3. ECFCs were incubated with 6.67 µM DiR, plated in ½ dilutions (from 200.000 cells to 781) and scanned on days 1, 2 and 3. **B** Standard curves (DiR intensity *vs* cell number) and linear equations obtained for days 1 and 3, corresponding to the averaged values of three independent standard curves performed. **C** Representative images obtained for DiR pre-labeled ECFCs (2·10^5^ and 1·10^5^) (top wells) and ECFCs (1·10^5^) incubated with the 3 consecutive washing solutions (1 ml PBS each) derived from the cells in the upper wells (bottom wells). **D** Scans from Labeled-ECFCs (2·10^5^ and 1·10^5^) (top) and ECFCs (1·10^5^) incubated with the remaining washing solutions (10 ml PBX 1X) from the, (bottom). **E** ECFCs (1·10^5^) were incubated with decreasing amounts of DiR (from 3,34 to 1.6·10^–3^ µM DiR, serial dilutions 1/2) **F** Standard curve and linear equation obtained after the incubation of ECFCs with DiR serial dilutions, corresponding to the averaged values of three independent standard curves performed. **G** Scanned plate with ECFCs (1·10^5^), unlabeled (DiR negative control), incubated with 6,67 µM DiR without washing or pre-incubated with 6.67 µM DiR after 3 washes (top row). ECFCs incubated with the supernatant from 3 consecutive washing solutions (lower row). **H** In vivo scans on day 0 (left) and day 1 (right) after administration of 0.2 µM DiR either IV (IV-) or IM (IM-), or administration of 10^6^ labeled ECFCs (6,67 µM DiR, IV + and IM +). **I** Ex vivo scans of spleen, kidneys, lungs, liver) and hind limbs, one day after injecting either DiR-labeled ECFCs or 0.2 µM DiR. A Negative control (with 10^6^ unlabeled ECFCs), was also included to discard background signals

The standard curve designed to determine the number of ECFCs based on DiR intensity values showed a clear correlation between cell number and DiR fluorescent signal (Fig. 2A). This relation remained with time, with R2 values > 0.99 during three consecutive days (Fig. 2B). Thereby, considering these results, these linear equations were applied later on to determinate cell numbers based on DiR intensity values.

#### Determination of the DiR residual staining

The DiR fluorescent signals of ECFCs stained with the residual solution derived from three consecutive washing steps (1 ml PBS 1X/wash) (Fig. 2C) were similar to the strong intensities registered for ECFCs (1·10^5^) directly labeled with standard protocols (direct labeling with 6.67 µM DiR solution and 3 washes with 1 ml PBS). By increasing the washing volume up to 10 ml PBS (Fig. 2D), a significant decrease of DiR signal was seen in ECFCs stained with the residual washing steps, detecting only 10% of the initial DiR seen in pre-labeled cells. In all cases, DiR intensities were proportional to cell numbers, showing half intensity values in the wells with half of cell numbers. Overall, these results indicated that the washes performed with 1 ml PBS 1X each were not sufficient to eliminate the residual DiR, and this could derive into false positive signals during in vivo biodistribution assays. By increasing the washing volume, we removed most of the residual DiR, so we fixed this protocol for future biodistribution assays.

#### Determination of the optimal residual concentration for DiR staining

According to the linear equation (R^2^:0.992) obtained between DiR-cell intensities and DiR concentrations (µM) (Fig. 2E-F), the fluorescence detected for ECFCs that did not undergo washing steps (Fig. 2G, second well, top row) correlated to 1.47 µM DiR, while the intensity of the same cells after the third wash (Fig. 2G, third well, top row) correlated to 1.13 µM DiR residual intensities. On the other hand, the DiR intensities of ECFCs incubated directly with the washing supernatants ranged from 0.34 µM (first wash) to 0.19 µM (third wash) (Fig. 2G, lower row), so we fixed 0.2 µM as the DiR residual solution to test in the in vivo assays.

Regarding the in vivo assays, a strong fluorescence signal was seen in mice injected with 10^6^ ECFCs stained with DiR (6.67 µM) (intravenously, IV + or intramuscularly, IM +), right after cell administration (Day 0) and on day 1 (Fig. 2H). On the other hand, the mouse injected intramuscularly with 0.2 µM residual DiR (IM-), also reported some signal in the calf muscle on days 0 and day 1. Finally, no fluorescence was detected in vivo after injecting the residual DiR solution intravenously (IV-), although some signals appeared in the spleen and the liver after the ex vivo scans (Fig. 2I). These results indicate that DiR can stain tissues even in the absence of cell administration. Therefore, biodistribution assays should include a negative control with DiR residual solution (apart from a negative control with unlabeled cells).

### DiR staining does not affect ECFCs viability or vessel formation properties

**Fig. 3:**
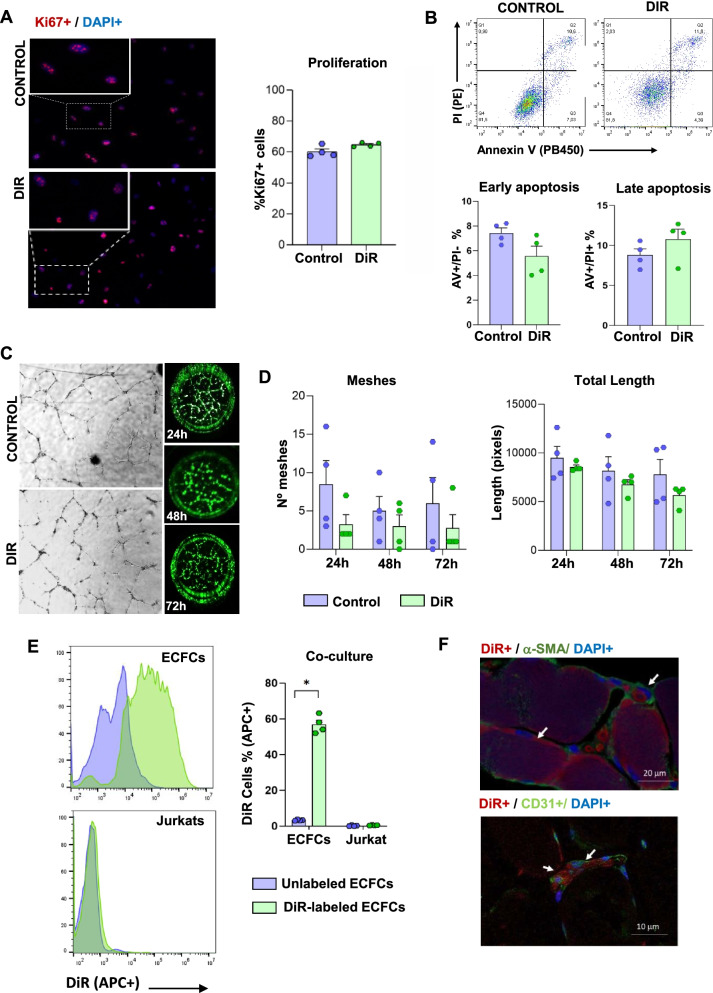
DiR labeling does not affect ECFCs functions. **A** Proliferation assays. Representative images of Ki67 + (red) unlabeled (control) and DiR-labeled ECFCs (DiR-ECFCs) (left). Percentage of Ki67 + cells of unlabeled and D- ECFCs (right). **B** Apoptosis assay. Representative dot-plots with Annexin V (AV) and propidium iodide (PI) from flow cytometry (top). Percentage of ECFCs and D-ECFCs in early (AV + /IP-, left) and late (AV + /IP + , right) apoptosis. **C** Angiogenesis assay**.** Representative images of matrigel tubule formation results, obtained with phase-contrast microscope (left) and NIR scanning (right). **D** Number of meshes quantified (left) and total tubule lengths (right) measured at 24 h, 48 h and 72 h, for unlabeled and DiR-labeled ECFCs. **E** Flow cytometry histograms obtained to detect DiR signal (APC +) after co-culturing 24 h DiR pre-labeled EFCFs and unlabeled Jurkats. Percentage of DiR + (APC +) cells detected for DiR-ECFCs and Jurkats after 24 h co-culture. Data were represented as the mean ± SEM indicating all independent values. *p-values < 0.05. **F** Representative IHC images confirming the presence of DiR labeled cells (red) within the tissue, 3 days after administration, colocalizing with human CD31 + (endothelial marker, green, left image) and also detected nearby blood vessels (*α*-SMA staining, green)

Functional assays confirmed that DiR staining did not affect ECFCs proliferation or viability (Fig. 3A–B). Similarly, ECFCs angiogenic potential was not affected as result of labeling them with DiR, as indicated in a matrigel tubule formation assay in vitro (Fig. 3C). The number of meshes and the total length of the tubules formed was similar in both, labeled and DiR labeled ECFCs, with no significant differences between them (Fig. 3D). Finally, while a strong signal was detected for DiR-labeled ECFCs by flow cytometry, no apparent dye transference from DiR-ECFCs to unlabeled Jurkats was seen after 24 h co-culture (Fig. 3E). In vivo assays confirmed the presence of DiR-labeled ECFCs within the limbs, corroborated by colocalization with human CD31 + staining. Moreover, DiR-labeled ECFCs were also found within the vessels (Fig. 3F).

### Biodistribution assays after ECFCs administration in a CLTI murine model.

#### Follow up of ECFCs by DiR labeling

**Fig. 4:**
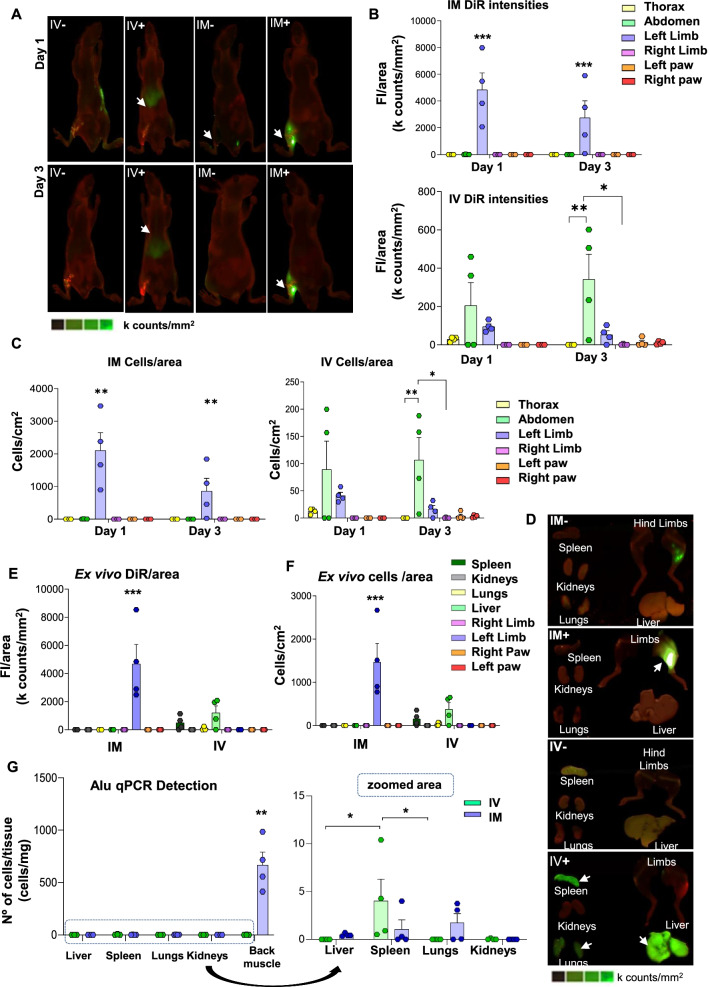
Biodistribution assay after ECFCs administration to CLTI mice. **A** In vivo scan on day 1 (top) and day 3 (bottom) after intramuscular (IM + , n:4) or intravenous (IV + , n:4) administration of 10^6^ DiR pre-labeled ECFCs to CLTI mice. A set of mice injected via tail vein (IV-, n:1) or intramuscular (IM-, n:1) with 0.2 µM DiR solution as residual DiR negative controls were included, together with a Negative Control (with 1·10^6^ unlabeled ECFCs) to consider background signals during the in vivo and ex vivo scans. **B** Fluorescence intensity (FI) per area (k counts/mm^2^) on days 1 and 3 after IM (top) or IV (bottom) injection of 1·10^6^ DiR pre-labeled ECFCs. **C** Cell number estimation per area (cells/cm^2^) of in vivo scans on days 1 and 3 of IM (left) and IV administration (right) ECFCs*.* **D** Ex vivo scans from spleen, kidneys, lungs, liver and hind limbs at day 3 after ECFCs IM or IV transplantation. **E** Fluorescence intensity (FI) per area (k counts/mm^2^) of organs after performing an ex vivo scan. **F** Cell number estimation per area (cells/cm^2^) of ex vivo scans. **G** Number of cells per tissue mg calculated after human-specific Alu gene amplification by qPCR in liver, spleen, lungs, kidneys and back muscle tissue at day 3

Next, we tested the optimized DiR labeling protocol with ECFCs administered either IM or IV to CLTI mice. As a result, one day after IM transplantation, DiR signal was mainly found in the injection area around the ischemic limb (*p*-value < 0.05 compared with the other areas except the abdomen). No emission was detected in other areas, such as thorax or abdomen (Figs. 4A–B). By day 3, the DiR-related intensity within the injection area decreased (*p*-value < 0.01), although no apparent translocation to other organs was seen (Figs. 4A–B).

Based on the calibration curve previously built, these intensities correlated with a high concentration of cells in the left limb after IM administration at day 1 (*p*-value < 0.05 compared to the rest areas except for the abdomen), and even more significantly at day 3 (*p*-value < 0.01, Fig. 4C). Finally, the ex vivo scans confirmed the localization of DiR fluorescence signal mostly in the left limb (*p*-value < 0.001 *vs* other tissues/organs) after IM administration.

On the other hand, the DiR signal from ECFCs administered intravenously (IV +) was mainly found in the abdominal area on day 1, followed by the left limb and thorax (Fig. 4A–C). At day 3, DiR intensity levels remained and even increased mainly in the abdomen (*p*-value < 0.01 *vs* the thoracic cavity, and *p*-value < 0.05 compared to the right limb) but decreased in the left limb and disappeared in the thoracic area. Finally, a slight fluorescence signal was found in left and right paws (Fig. 4C). The ex vivo scans of IV transplanted mice revealed that the abdomen fluorescence came from the liver and spleen, and a faint emission was also observed in lungs (Fig. 4E–F).

### qPCR analysis of ECFCs biodistribution

Complementary to the DiR cell-tracking assay, qPCR data confirmed the presence of ECFCs mainly in the back left muscle, 3 days after IM injection (668.60 ± 122.11 ECFCs/tissue mg; *p*-value < 0.01), but also traces of hDNA in lungs, spleen and liver (1.72 ± 0.99; 1.08 ± 0.98; 0.48 ± 0.08; respectively) (Fig. 4G). On the other hand, hDNA was detected mainly in the spleen after IV injection (4.03 ± 2.29 ECFCs per tissue mg; p-value < 0.05 compared with liver and lungs) and in lower quantity in the back muscles adjacent to the femoral artery (1.82 ± 0.99 ECFCs/tissue mg) (Fig. 4G).

## Discussion

We and other researchers have already described the beneficial effects of cell therapy in murine models of CLTI, mainly by promoting blood flow perfusion recovery after enhancement of collateral vessel formation [33, 42, 43]. Similarly, the therapeutic effects of endothelial progenitor cells (EPCs) transplantation in different models of ischemia have already been described [11, 12, 44–46], and the potential use of ECFCs to promote revascularization in CLTI patients seems undeniable [47–49]. These cells enhance revascularization by promoting vessel formation and perfusion recovery, alone or in combination with MSCs [8, 50, 51]. Besides, some preclinical studies have already reported a long-term presence of injected ECFCs within the ischemic area, without provoking any adverse effects [12, 51].

Before any potential clinical translation of these cells, several assays are required by regulatory authorities to ensure the safety and efficacy of these potential therapeutic agents [13, 14]. Similarly, other issues as the optimal route of administration and cell biodistribution following transplantation are important concerns to be evaluated. For instance, the delivery route might depend on the cell type or cell dose, and the chosen implantation strategy can significantly affect the therapeutic success [52, 53].

To date, different studies have analyzed the potential of using IM implantation as an alternative to IV in preclinical [52, 54] and clinical trials including CLTI patients (i.e., NCT03968198, NCT02993809, NCT04466007; www.clinicaltrials.gov). IM implantation has been also compared to intraarterial (IA) cell administration in several clinical studies, presenting similar and/or complementary effects [55–57]. However, most studies targeting CLTI have relied on IM transplantation, most probably because it is less invasive and easier than IA administration [58]. Besides, only a few preclinical assays have been able to reproduce IA administration in animal models [59], mainly due to the difficulty of this approach in small animals.

Herein, we have evaluated, for the first time to our knowledge, which route, either IM or IV administration of ECFCs, could derive in a more focalized delivery of these cells in a CLTI murine model. Our in vivo and ex vivo DiR and qPCR tracking approach confirmed that after IM administration, ECFCs were retained in the ischemic area, where they were injected, in agreement with other studies using these or other cell types [52, 60–62]. Also, although the DiR assays did not report any apparent translocation to any other organs or tissues, qPCR results reported negligible numbers of hcells in lungs, spleen and liver. We and others have previously described how cells can migrate to the vascular vessels even after IM delivery. Thus, ECFCs might have gone into circulation until the reaching of distal organs, as suggested [63–65].

On the other hand, DiR labeled ECFCs administered intravenously were detected around the abdominal cavity, less intensively in the left limb and barely in the thorax. The abdominal signal came mainly from the liver and spleen, with some fluorescence also detected in the lungs. These data confirmed the necessity to perform ex vivo monitoring together with the in vivo cell tracking, in order to avoid signal loss associated with the interference of epithelial layers [59, 66, 67]. Finally, qPCR results corroborated the presence of human ECFCs mainly in the spleen after IV injections, as previously reported [68], and low levels in the liver, where other researchers also found IV-administered endothelial cells [59, 68]. Similarly, cells like bone marrow stromal cells or MSCs are known to accumulate in the lungs when administered IV, with potentially adverse effects [69, 70].

Thus, while health authorities should rule the acceptable number of cells that could be present in nontarget organs, our data corroborated the importance of conducting these approaches to determine the best administration route for a specific cell therapy candidate, prior to any potential translation into the clinic.

Our results also highlighted the importance to complement imaging studies with DNA analysis, to confirm and support imaging-based biodistribution assays [30, 71]. Regarding the two approaches selected here, DiR in vivo cell tracking or post-mortem qPCR analysis, they both have their advantages and disadvantages. DiR cell tracking resulted in a successful strategy to provide in vivo biodistribution of ECFCs with dependence on the delivery route. Moreover, DiR staining did not affect cell proliferation or viability or their capability to form vessels. Yet, it seems compulsory to consider that although in small extent, probe release or dilution during cell division, cell death or phagocytic events could cause false positive signals. Conversely, qPCR analysis does not provide information “in vivo”, but quantification of hDNA using specific Alu sequences has resulted in a highly sensitive approach, with a detection range of 1 human cell in 10,000 mice cells.

Finally, we have shown that, despite many researchers reassuring that DiR does not promote unspecific staining, residual DiR (released during the cell labeling protocol) can significantly stain cells and tissues. This fact can remarkably affect the estimation of cell biodistribution and the determination of cell quantity based solely on DiR staining if the effect of un-appropriate labeling protocol (i.e., washing steps) is not considered. Our results indicate that the volume of the washing solution is crucial, with intense signals detected in cells after following the standard procedure applied for DiR labeling. Indeed, the residual dye could still stain cells in similar levels than the original DiR labeling concentration. Thus, astringent washing steps are required to ensure the optimal removal of any residual DiR dye. Likewise, in vivo studies including DiR-cell staining have often used mice injected with unlabeled cells as negative controls, without taking into account that DiR alone provides a strong signal by itself. Although we did not detect transference between co-cultured cells in vitro, we reported fluorescence in mice injected (IM or IV) with the residual solution (0.2 µM DiR), suggesting that a residual DiR negative control should be included in order to discard any false positive signals.

## Conclusion

Herein, we describe an optimized workflow for an accurate in vivo and ex vivo cell tracking analysis with DiR, a NIR lipophilic labeling dye, for its use in preclinical biodistribution assays. Overall, intensive washing steps and residual DiR negative controls are required in order to properly quantify cells based on DiR labeling, to avoid false positive signals coming from the dye itself, both in vivo and ex vivo. Also, in vivo scans do not provide accurate information regarding the fluorescence intensity signals associated to internal organs, so ex vivo scans are necessary to confirm the presence and quantity of cells within the organs of interest. Besides, our results indicate that a combination of in vivo DiR imaging with qPCR amplification of human DNA constitutes a highly sensitive approach to track cells systemically.

We have shown that human ECFCs can be efficiently labeled with DiR and tracked in vivo and ex vivo in immunosuppressed mice. Moreover, according to our results, ECFCs administrated intramuscularly remained mainly within the ischemic limb while those injected intravenously were found in several organs, mostly in the spleen and some traces in the liver. While future studies are warranted to determine the precise durability of the administered DiR labeled cells within the limbs, our data support intramuscular administration of ECFCs in CLTI mice as an optimal strategy to ensure a more focalized distribution and therefore higher availability of the transplanted cells in the ischemic area. Our data support the requirement of biodistribution studies before any potential translation of cell-based therapy approaches into the clinic, in order to obtain an overview of possible and undesirable cellular translocations outside the areas of interest.

## Supplementary Information

**Additional file 1.** Supplementary Materials. Supplementary Figure S1. Supplementary Tables S1–S12.

## Data Availability

All data generated or analyzed during this study are included in this published article, and its supplementary information files.

## Abbreviations

AV: Annexin V
CLTI: Critical limb-threatening ischemia
DiR: 1,1-Dioctadecyl 3,3,3,3 tetramethylindotricarbocyanine iodide
ECFCs: Endothelial colony-forming cells
hAlu: Human-specific Alu
hcells: Human cells
hDNA: Human DNA
IM: Intramuscular
IV: Intravenous
MSCs: Mesenchymal stem cells
NIR: Near-infrared
PFA: Paraformaldehyde
PI: Propidium iodide
*α*-SMA: *α*–Smooth muscle actin
