# Supplementary material for: Assessment of endothelial colony forming cells delivery routes in a murine model of critical limb threatening ischemia using an optimized cell tracking approach
Source: Stem Cell Res Ther. 2022 Jun 21;13:266. doi: 10.1186/s13287-022-02943-8 (PMC9210810; doi:10.1186/s13287-022-02943-8)
Supplement: Supplementary file 1 — Additional file 1. Supplementary Materials. Supplementary Figure S1. Supplementary Tables S1–S12. [file 13287_2022_2943_MOESM1_ESM.docx]

**ASSESSMENT OF ENDOTHELIAL COLONY FORMING CELLS DELIVERY ROUTES IN CRITICAL LIMB THREATENING ISCHEMIC MICE WITH AN OPTIMIZED CELL TRACKING APPROACH**

**SUPPLEMENTARY MATERIALS**

**Supplementary Figure S1**


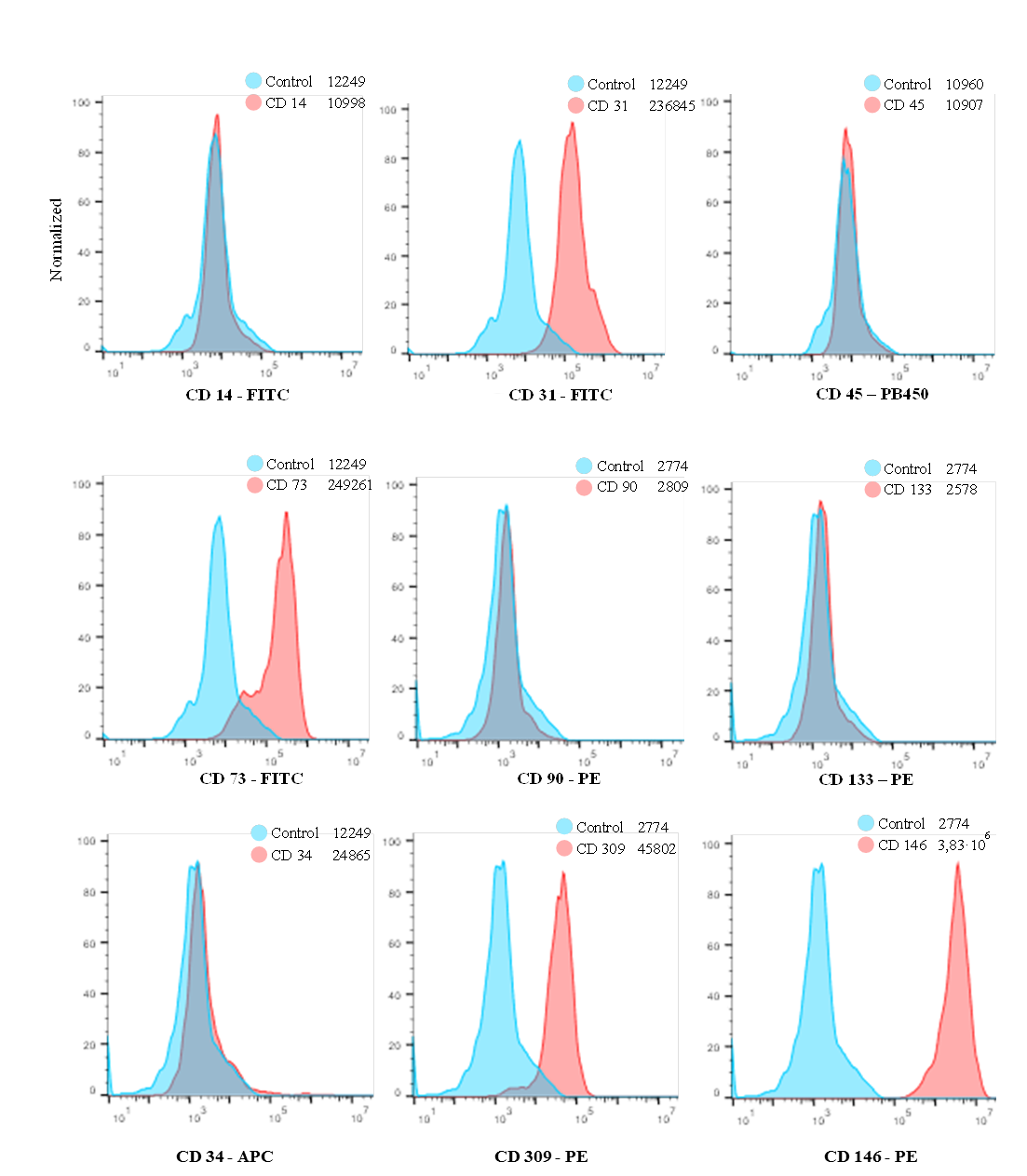


CD14 - FITC

CD31 - FITC

CD45 – PB450

Control 12249

CD14 10998


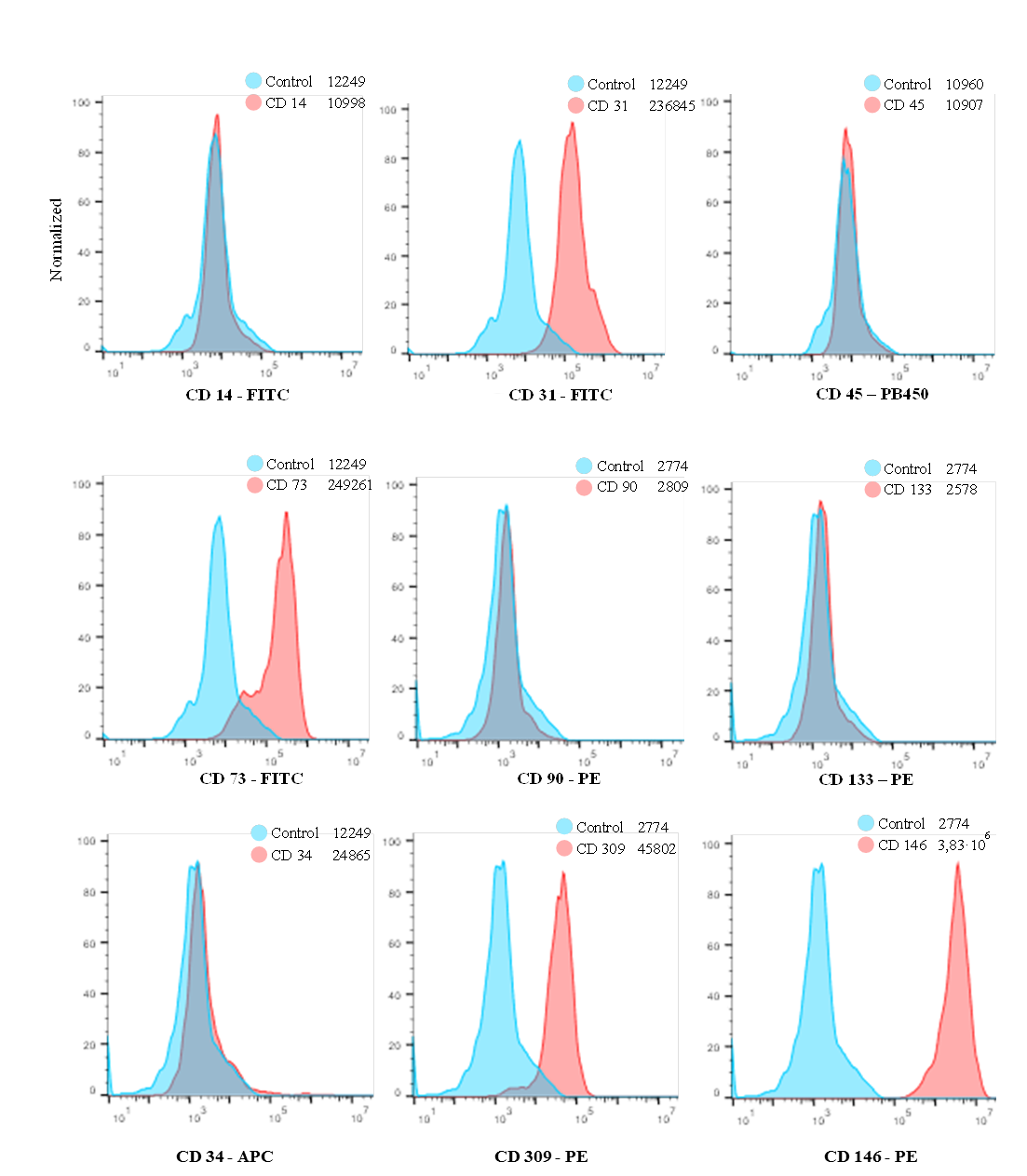


Control 12249

CD31 236845


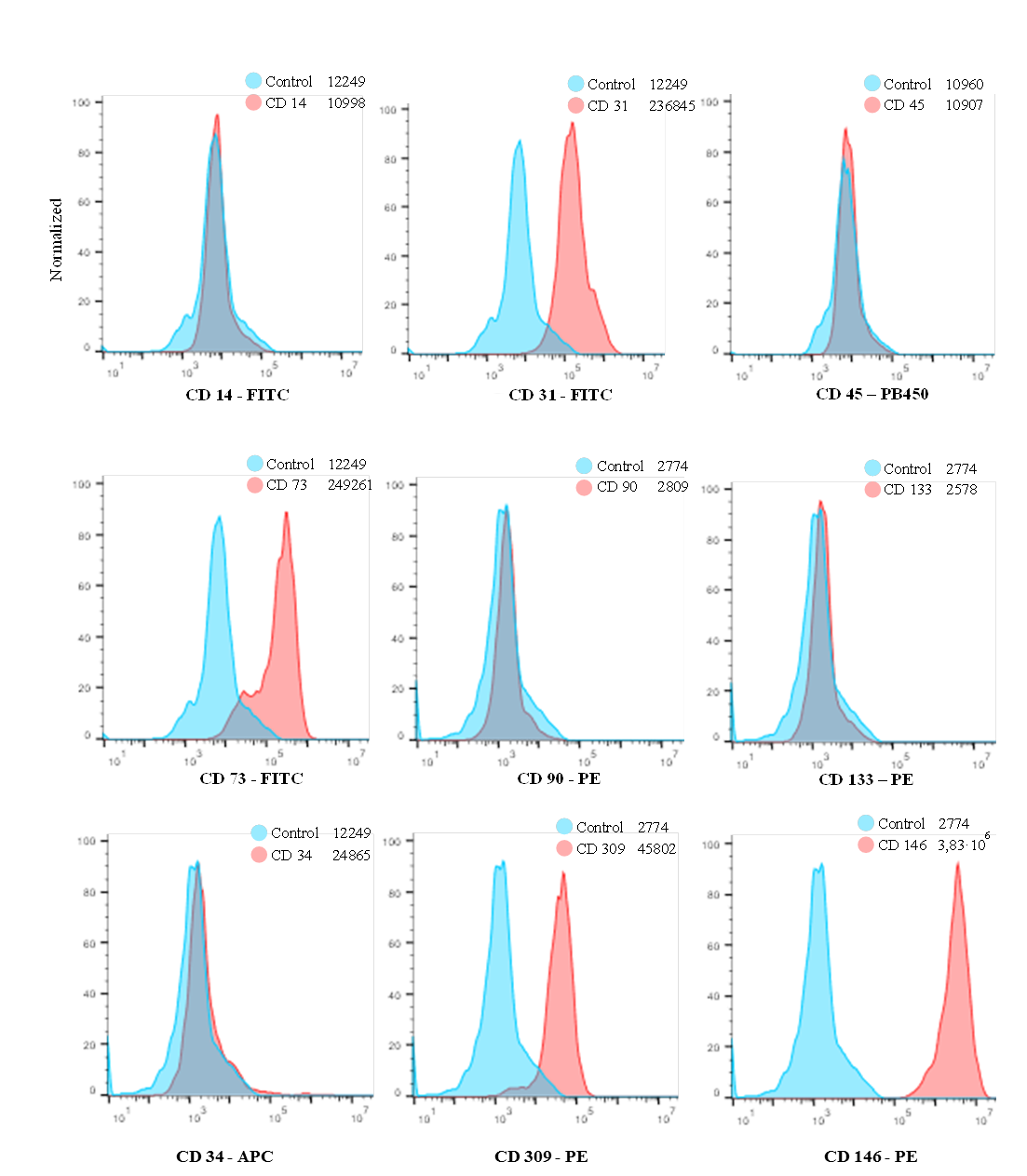


Control 10960

CD45 10907


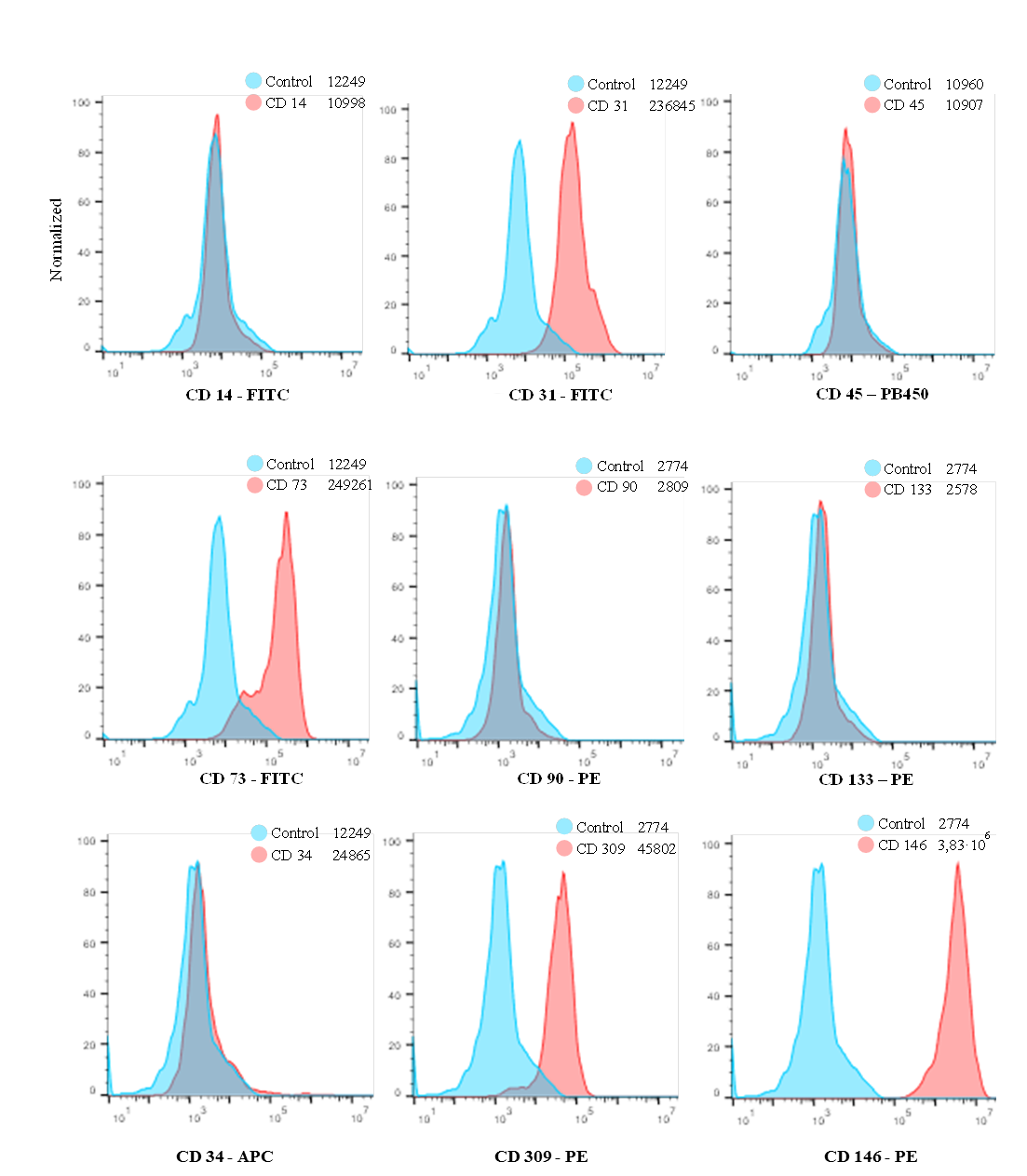


CD73 - FITC

Control 12249

CD73 249261


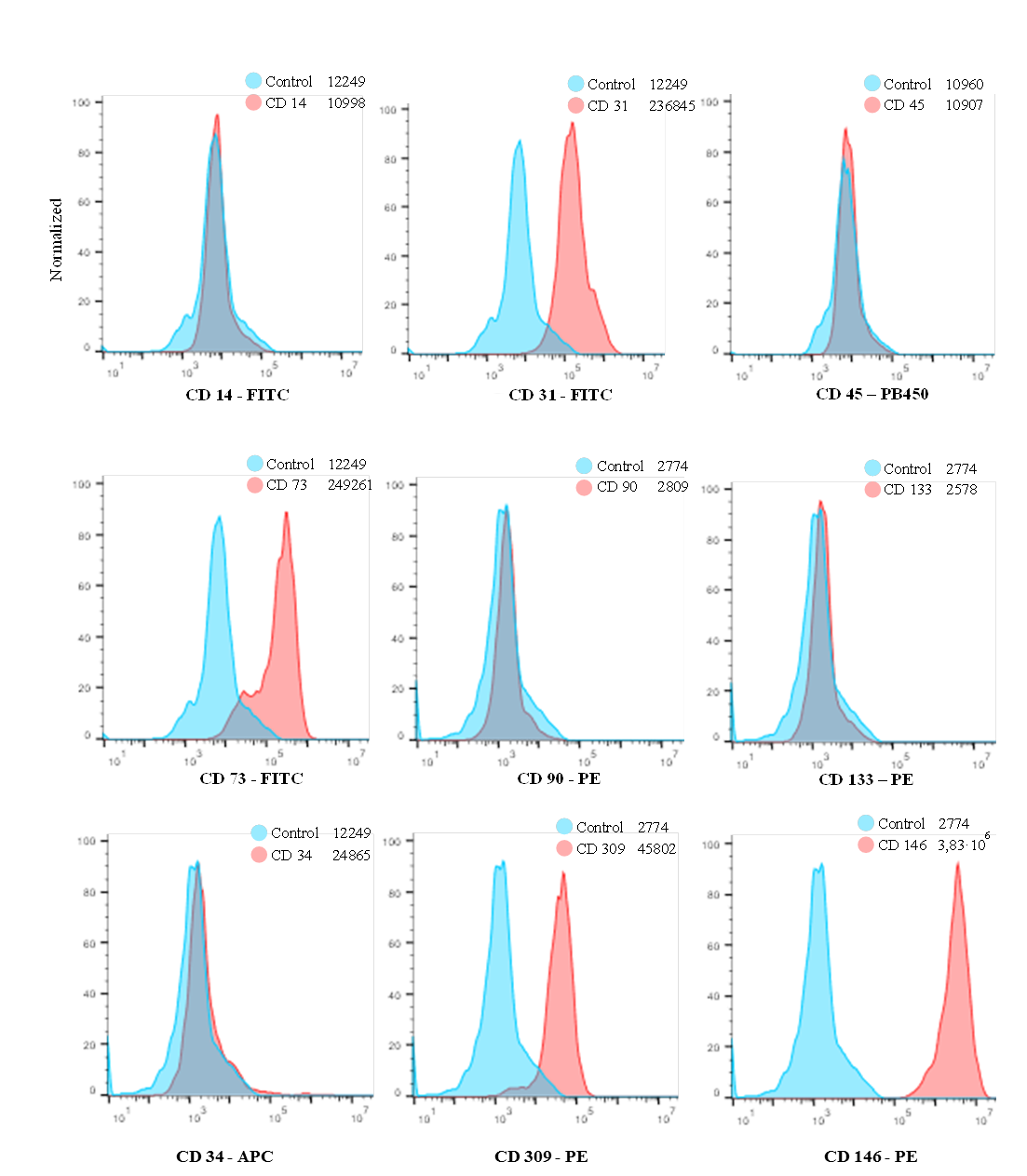


Control 2774

CD90 2809


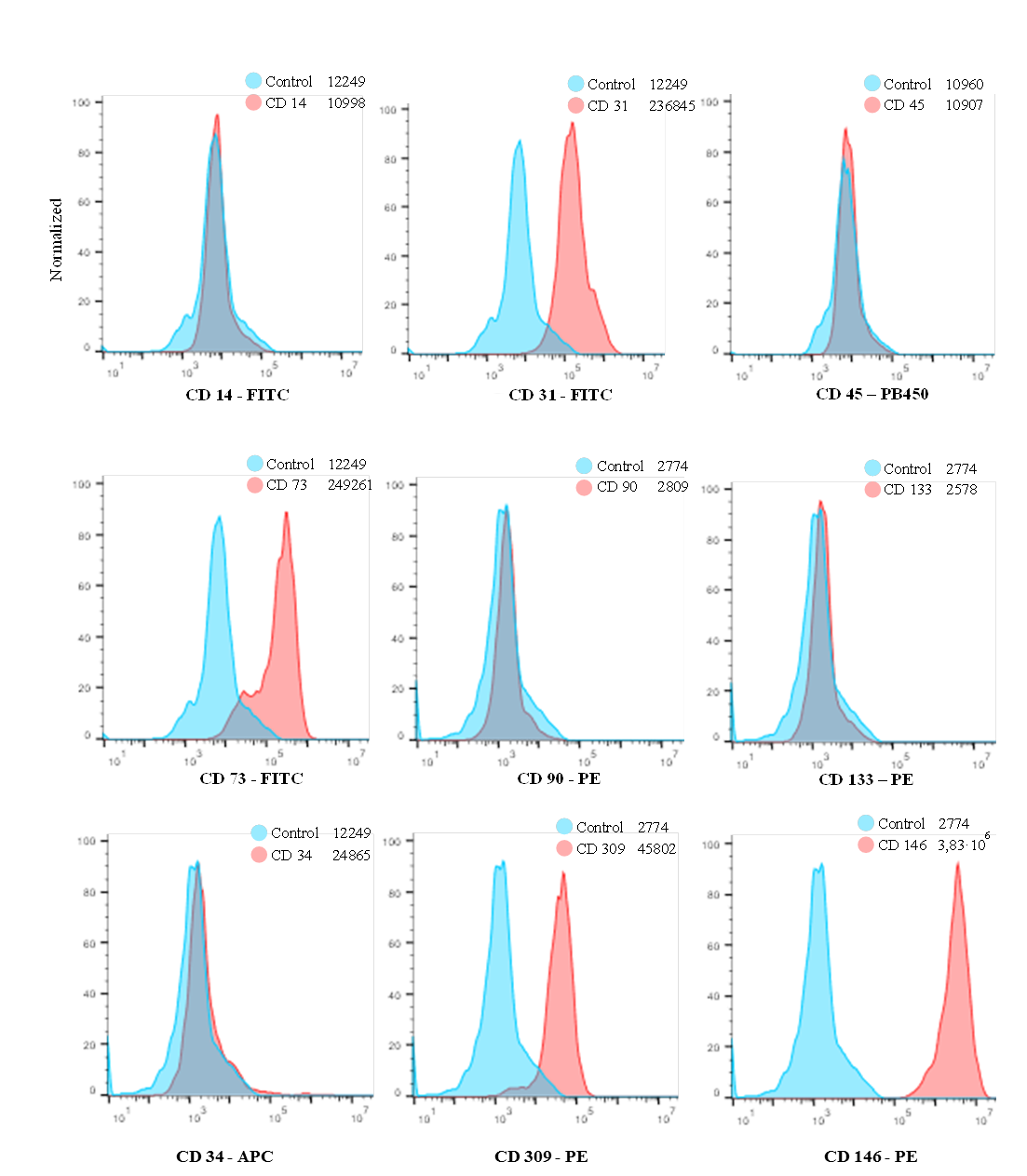


Control 2774

CD133 2578


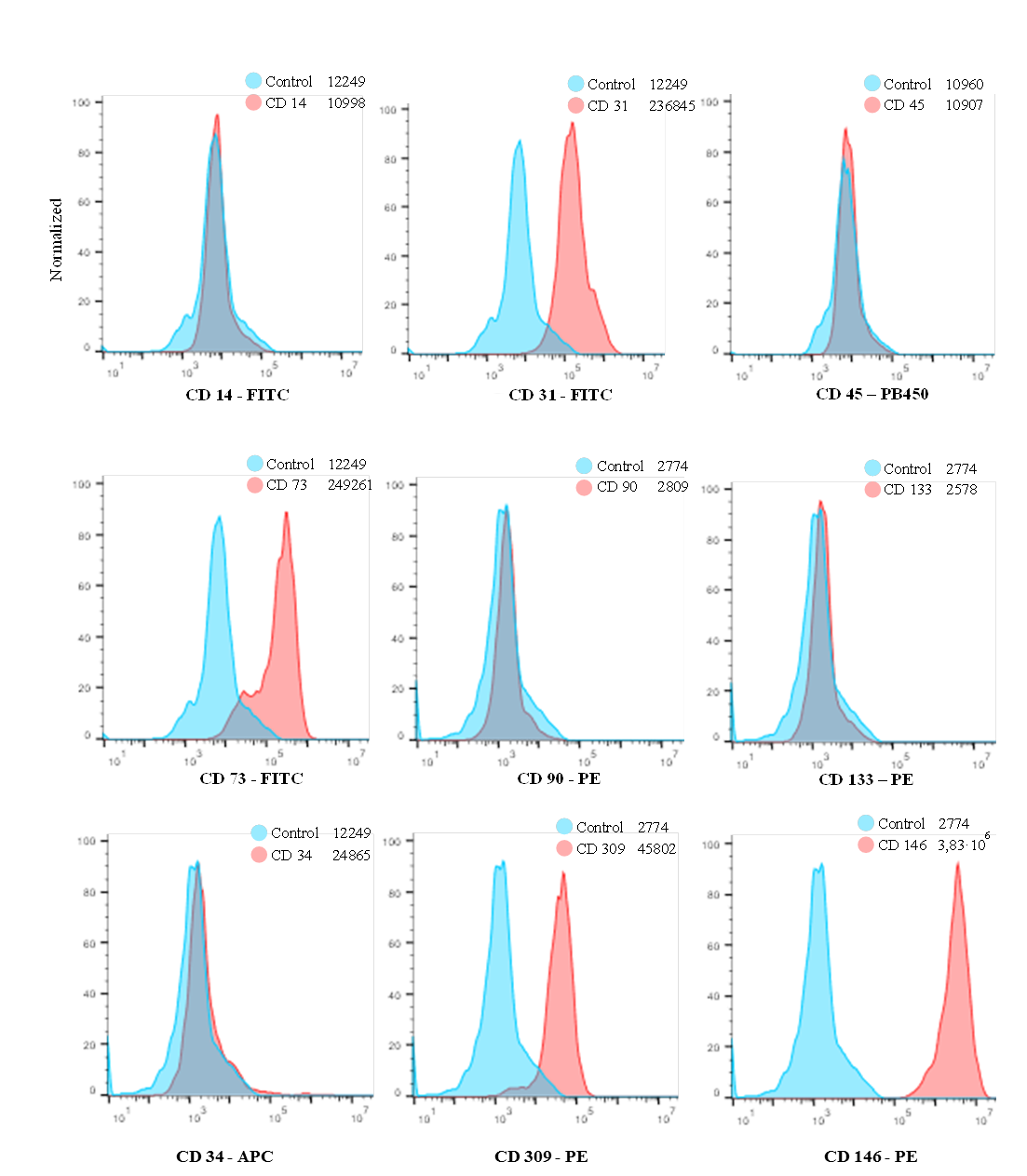


CD90 - PE

CD133 - PE

CD34 - APC

Control 12249

CD34 24865


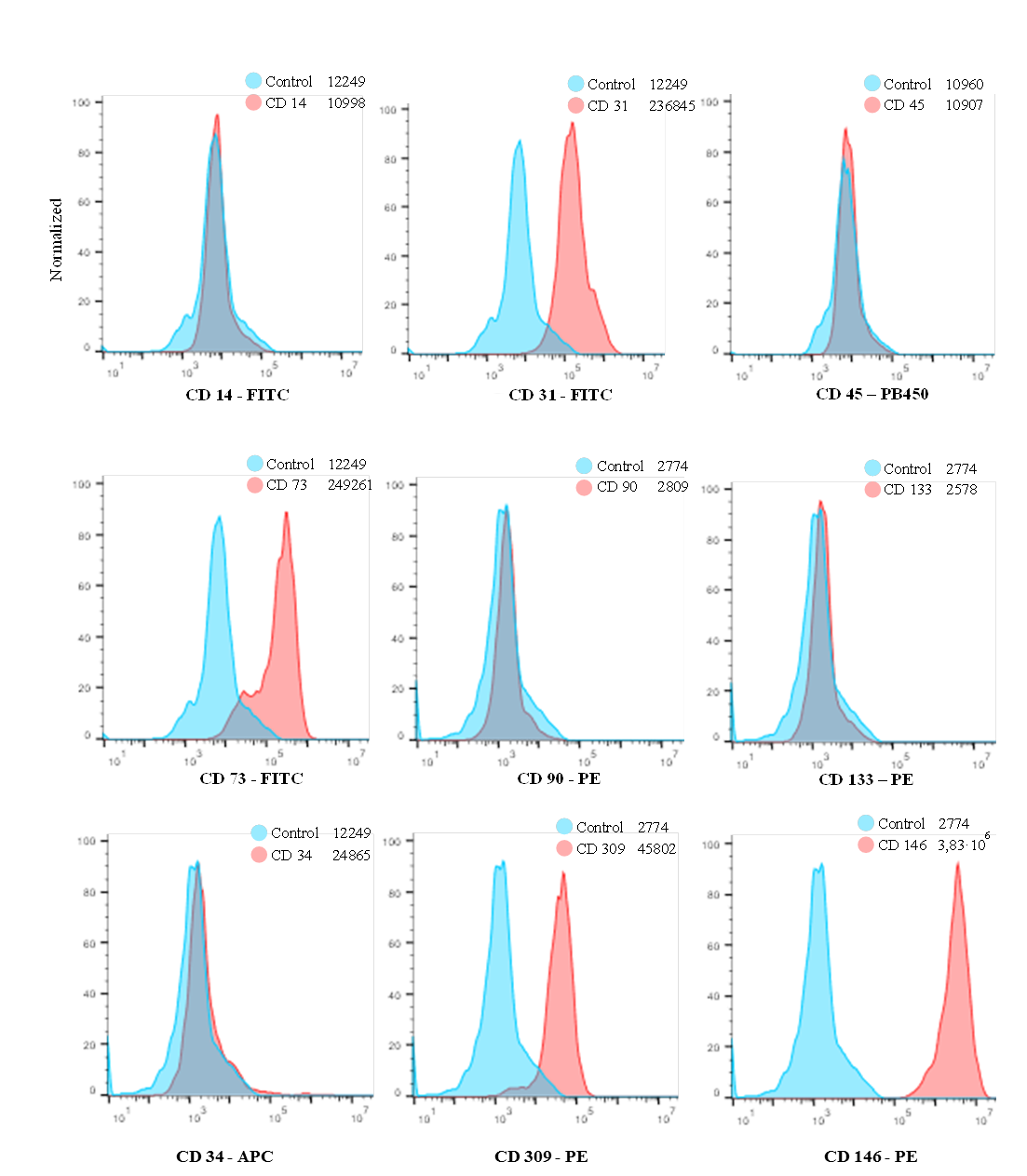


CD309 - PE

Control 2774

CD309 45802


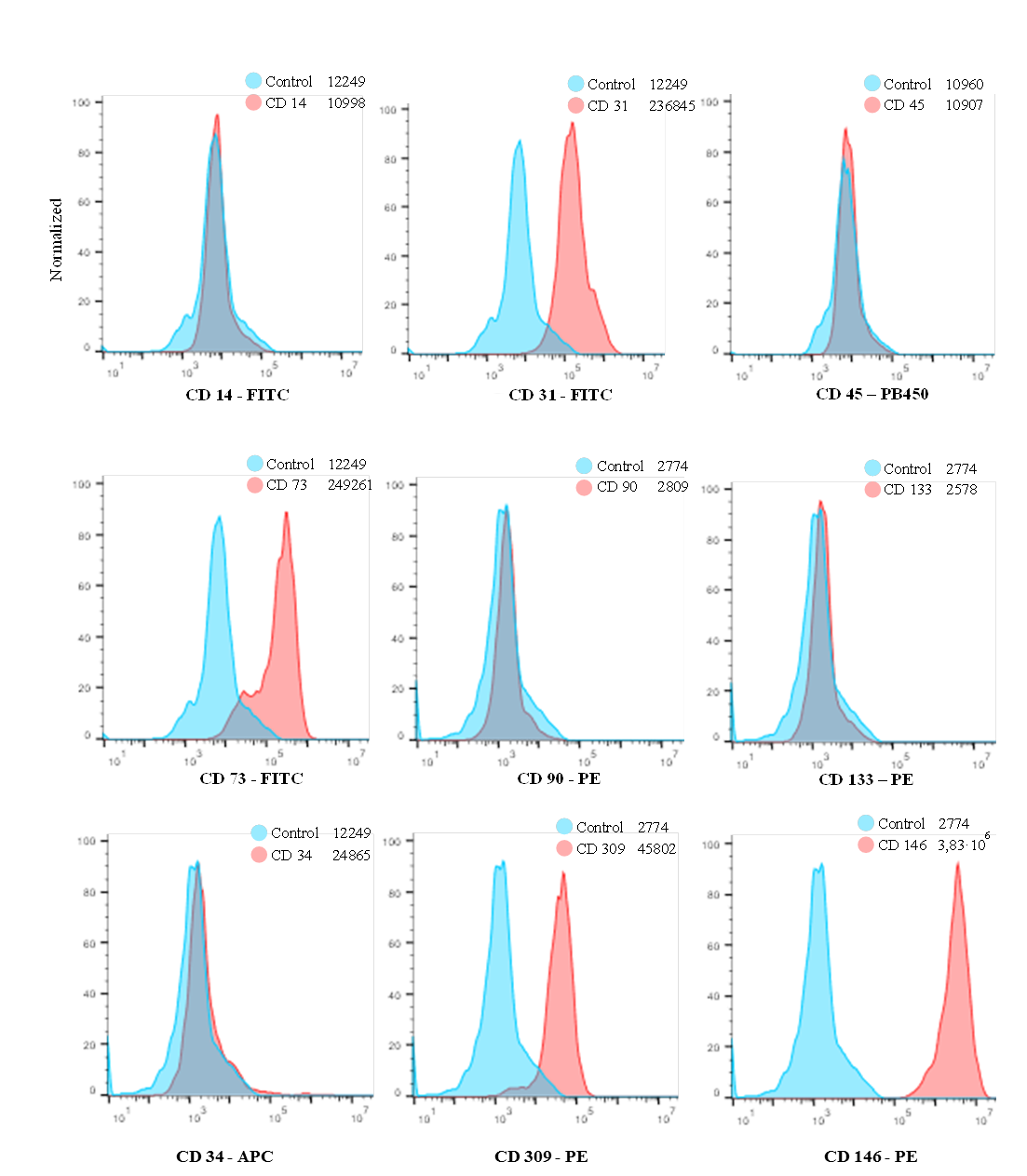


CD146 - PE

Control 2774

CD146 3,83·10^6^


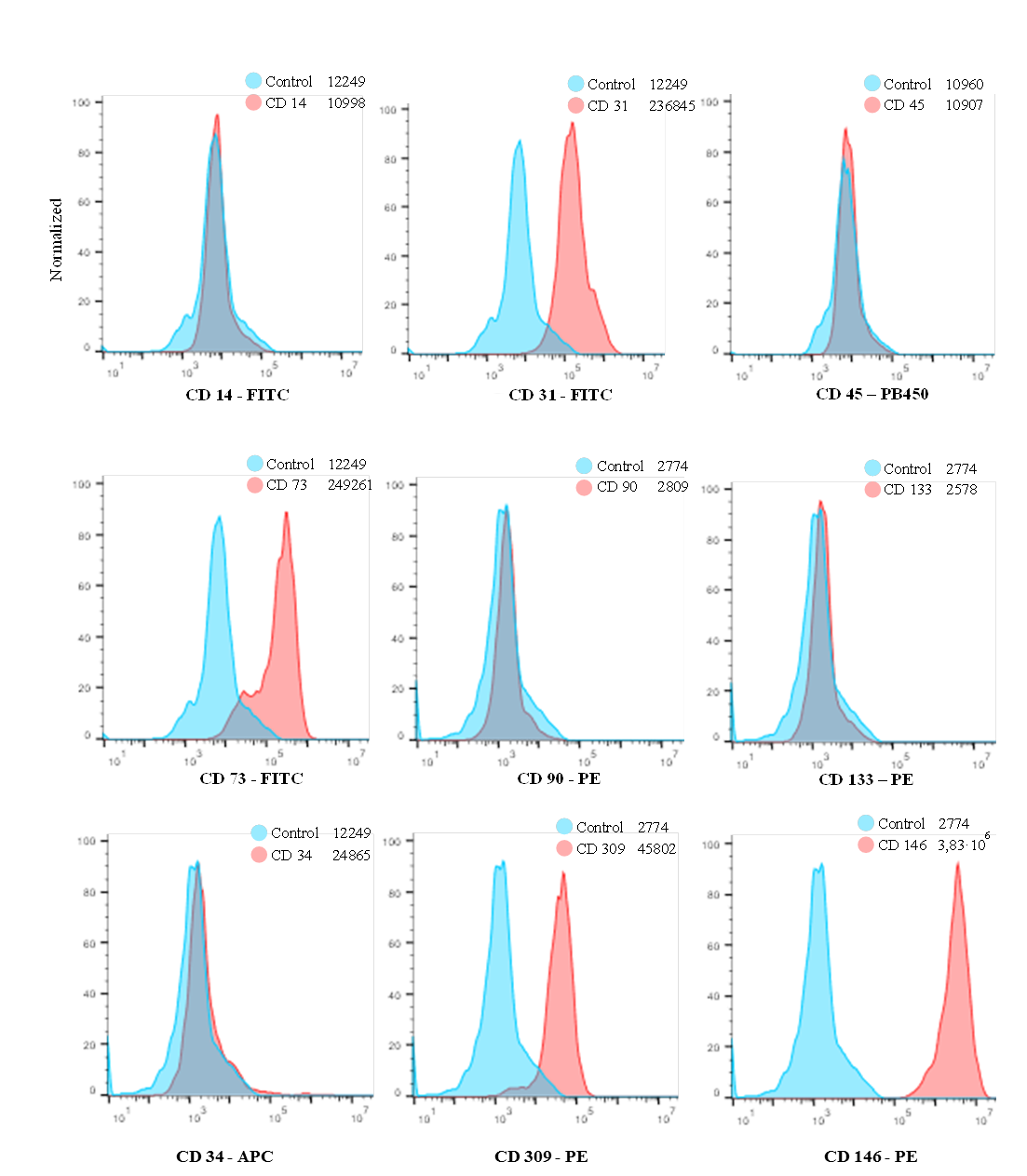


Normalized

**Figure S1. Characterization of ECFCs.** Cell identity was confirmed by flow cytometry, analyzing the following markers: CD31, CD34, CD45, CD90, CD73, CD309 (VEGFR2), CD133, CD146 and CD14. Specific isotype antibodies were used as negative controls. Data were presented as Mean Fluorescence Intensity (MIF), for both, the isotype control (blue) and positive markers analyzed (red).

**Supplementary Tables**

**Table S1. Primary antibodies employed in this study.** H: Human; M: Mouse; R: Rat; FC: Flow cytometry; IC: Immunochemistry; IHC: Immuhistochemistry.

| **Antibody** | **Reactivity** | **Dilution** | **Supplier** | **Reference** | **Used in** |
| --- | --- | --- | --- | --- | --- |
| CD14-PB | H | 1:25 | Biolegend | #367121 | FC |
| CD31-FITC | H | 1:25 | Biolegend | #303103 | FC |
| CD34-APC | H | 1:25 | Biolegend | #343607 | FC |
| CD45-PB | H | 1:25 | Biolegend | #368539 | FC |
| CD73-FITC | H | 1:25 | Biolegend | #303103 | FC |
| CD90 (Thy1)-PE | H | 1:25 | Biolegend | #328109 | FC |
| CD133-PE | H | 1:25 | Miltenyi Biotec | 130-098-826 | FC |
| CD146-PE | H | 1:25 | Biolegend | #361005 | FC |
| CD309-PE | H | 1:25 | Biolegend | #359903 | FC |
| IgG1 isoptype | H | 1:25 | Becton-Dickinson | 345816 | FC |
| α-Ki67 antibody | H,R | 1:500 | Invitrogen | PA5-16785 | IC |
| CD3-PE | H | 1:25 | Biolegend | #317308 | FC |
| α-smooth muscle actin | M | 1:500 | Sigma | A5228 | IHC |
| CD31 | H | 1:500 | Abcam | Ab32457 | IHC |

**Secondary antibodies used in this study.** Rb: Rabbit; IC: Immunochemistry.

| **Antibody** | **Reactivity** | **Dilution** | **Supplier** | **Reference** | **Used in** |
| --- | --- | --- | --- | --- | --- |
| Alexa Fluor 555 | Rb | 1:500 | ThermoFisher | A-21428 | IC |
| Alexa Fluor 488 | Rb | 1:500 | ThermoFisher | A11008 | IHC |
| Alexa Fluor 488 | M | 1:500 | ThermoFisher | A10667 | IHC |

**Table S2.** Intensity of the fluorescence emission after labelling both 2·10^5^ and 10^5^ ECFCs with 6,67 μM DiR dye. Then, cells were washed three times with 1 ml or 10 ml PBS and another set of 105 ECFCs were incubated with the different supernatant solution collected after centrifugation.

|  | **Washing steps of 1ml** | **Washing steps of 10ml** |
| --- | --- | --- |
| **2·10^5^ ECFCs** | 599.54 | 559.47 |
| **1·10^5^ ECFCs** | 270.57 | 255.29 |
| **1^st^ supernatant** | 242.45 | 35.99 |
| **2^nd^ supernatant** | 296.00 | 25.01 |
| **3^rd^ supernatant** | 199.22 | 20.58 |

**Table S3.** **Fluorescence intensity emission per area (k counts/mm^2^)** following DiR ECFCs labeling and NIR *in vivo* imaging in Balb-c nude mice administered via intramuscularly (IM) or intravenously (IV) at days 1 and 3 post-injection. Mean intensities and SEM are shown for the six areas selected.

|  | **IM** | | | | **IV** | | | |
| --- | --- | --- | --- | --- | --- | --- | --- | --- |
|  | **Day 1** | | **Day 3** | | **Day 1** | | **Day 3** | |
|  | **Mean** | **SE** | **Mean** | **SE** | **Mean** | **SE** | **Mean** | **SE** |
| **Thorax** | 0.00 | 0.00 | 0.00 | 0.00 | 29.17 | 5.62 | 0.00 | 0.00 |
| **Abdomen** | 12.77 | 7.58 | 0.00 | 0.00 | 205.13 | 120.14 | 341.40 | 131.33 |
| **Left Limb** | 4838.16 | 1256.89 | 2743.70 | 1266.96 | 95.16 | 13.66 | 52.21 | 21.54 |
| **Right Limb** | 0.00 | 0.00 | 0.00 | 0.00 | 0.00 | 0.00 | 1.49 | 1.49 |
| **Left Paw** | 0.00 | 0.00 | 0.00 | 0.00 | 0.00 | 0.00 | 12.27 | 10.79 |
| **Right Paw** | 0.00 | 0.00 | 0.00 | 0.00 | 0.00 | 0.00 | 10.36 | 4.03 |

**Table S4.** **Equivalence in number of cells per area (nºcells/mm^2^)** following DiR ECFCs labeling and NIR *in vivo* imaging in Balb-c nude mice administered via intramuscularly (IM) or intravenously (IV) at days 1 and 3 post-injection. Mean values and corresponding SEM are shown for the six areas selected.

|  | **IM** | | | | | | | **IV** | | | | | |
| --- | --- | --- | --- | --- | --- | --- | --- | --- | --- | --- | --- | --- | --- |
|  | **Day 1** | | | | **Day 3** | | | **Day 1** | | | **Day 3** | |  |
|  | **Mean** | | **SE** | | **Mean** | **SE** | | **Mean** | | **SE** | **Mean** | **SE** |  |
| **Thorax** | | 0.00 | | 0.00 | 0.00 | | 0.00 | 12.68 | 2.44 | | 0.00 | 0.00 | |
| **Abdomen** | | 5.55 | | 3.30 | 0.00 | | 0.00 | 89.19 | 52.24 | | 106.69 | 41.04 | |
| **Left Limb** | | 2103.55 | | 546.47 | 857.40 | | 395.93 | 41.37 | 5.94 | | 16.32 | 6.73 | |
| **Right Limb** | | 0.00 | | 0.00 | 0.00 | | 0.00 | 0.00 | 0.00 | | 0.46 | 0.46 | |
| **Left Paw** | | 0.00 | | 0.00 | 0.00 | | 0.00 | 0.00 | 0.00 | | 3.84 | 3.37 | |
| **Right Paw** | | 0.00 | | 0.00 | 0.00 | | 0.00 | 0.00 | 0.00 | | 3.24 | 1.26 | |

**Table S5.** **DiR fluorescence intensity emission per area (k counts/mm^2^) and equivalence in nº of cells per area (nºcells/mm^2^) following DiR ECFCs labeling and NIR *ex vivo* imaging.** Data are presented as mean±se for the areas indicated, in CLTI mice receiving ECFCs intramuscular (IM) and intravenously (IV), on days 1 and 3 after ECFCs administration.

|  | **IM** | | | | **IV** | | | |
| --- | --- | --- | --- | --- | --- | --- | --- | --- |
|  | **Fluorescence intensisty/area** | | **Number of cells/area** | | **Fluorescence intensisty/area** | | **Number of cells/area** | |
|  | **Mean** | **SEM** | **Mean** | **SEM** | **Mean** | **SEM** | **Mean** | **SEM** |
| **Spleen** | 0.00 | 0.00 | 0.00 | 0.00 | 494.30 | 254.55 | 154.47 | 79.55 |
| **Kidneys** | 0.00 | 0.00 | 0.00 | 0.00 | 0.00 | 0.00 | 0.00 | 0.00 |
| **Lungs** | 0.00 | 0.00 | 0.00 | 0.00 | 114.42 | 62.52 | 35.76 | 19.54 |
| **Liver** | 0.00 | 0.00 | 0.00 | 0.00 | 1201.24 | 498.04 | 375.39 | 155.64 |
| **Left Limb** | 4690.19 | 1381.97 | 1465.68 | 431.87 | 0.00 | 0.00 | 0.00 | 0.00 |
| **Right Limb** | 0.00 | 0.00 | 0.00 | 0.00 | 0.00 | 0.00 | 0.00 | 0.00 |
| **Left Paw** | 0.00 | 0.00 | 0.00 | 0.00 | 0.00 | 0.00 | 0.00 | 0.00 |
| **Right Paw** | 0.00 | 0.00 | 0.00 | 0.00 | 0.00 | 0.00 | 0.00 | 0.00 |

**Table S6**. **Statistical p-values related to fluorescence intensity/area following IM injection of DiR-labeled ECFCs and NIR imaging.** A Kruskal-Wallis test and Dunn’s test as post hoc analysis were applied to compare fluorescence intensity/area per region of interest, in Balb-c mice transplanted with ECFCs one day (orange) and three days (blue) after administration.

|  | **Thorax** | **Abdomen** | **Left Limb** | **Right Limb** | | **Left Paw** | | **Right Paw** | |
| --- | --- | --- | --- | --- | --- | --- | --- | --- | --- |
| **Thorax** | - | >0.9999 | 0.0033 | >0.9999 | | >0.9999 | | >0.9999 | |
| **Abdomen** | 0.2575 | - | 0.0033 | >0.9999 | | >0.9999 | | >0.9999 | |
| **Left Limb** | 0.0127 | >0.9999 | - | 0.0033 | | 0.0033 | | 0.0033 | |
| **Right Limb** | >0.9999 | 0.2575 | 0.0127 | - | >0.9999 | | >0.9999 | |  |
| **Left Paw** | >0.9999 | 0.2575 | 0.0127 | >0.9999 | - | | >0.9999 | |  |
| **Right Paw** | >0.9999 | 0.2575 | 0.0127 | >0.9999 | >0.9999 | | - | |  |

**Table S7.** **Statistical p-values related to fluorescence intensity/area after IV injection of DiR-labeled ECFCs and NIR imaging.** A Kruskal-Wallis test and Dunn’s test as post hoc analysis were applied to compare fluorescence intensity/areas (k counts/mm^2^) per area of interest, in Balb-c mice transplanted with ECFCs one day (orange) and three days (blue) after administration.

|  | **Thorax** | **Abdomen** | **Left Limb** | **Right Limb** | **Left Paw** | **Right Paw** |
| --- | --- | --- | --- | --- | --- | --- |
| **Thorax** | - | 0.0080 | 0.1407 | >0.9999 | >0.9999 | 0.8920 |
| **Abdomen** | >0.9999 | - | >0.9999 | 0.0432 | 0.5178 | >0.9999 |
| **Left Limb** | >0.9999 | >0.9999 | - | 0.5178 | >0.9999 | >0.9999 |
| **Right Limb** | 0.6669 | >0.9999 | 0.0555 | - | >0.9999 | >0.9999 |
| **Left Paw** | 0.6669 | >0.9999 | 0.0555 | >0.9999 | - | >0.9999 |
| **Right Paw** | 0.6669 | >0.9999 | 0.0555 | >0.9999 | >0.9999 | - |

**Table S8**. Statistical p-values obtained after performing a Kruskal Wallis test and Dunn’s test as post hoc analysis of the number of cells per area, at day 1 (orange) and day 3 (blue) after IM injection of DiR-labeled ECFCs.

|  | **Thorax** | **Abdomen** | **Left Limb** | **Right Limb** | **Left Paw** | **Right Paw** |
| --- | --- | --- | --- | --- | --- | --- |
| **Thorax** | - | >0.9999 | 0.0033 | >0.9999 | >0.9999 | >0.9999 |
| **Abdomen** | 0.2575 | - | 0.0033 | >0.9999 | >0.9999 | >0.9999 |
| **Left Limb** | 0.0127 | <0.9999 | - | 0.0033 | 0.0033 | 0.0033 |
| **Right Limb** | >0.9999 | 0.2575 | 0.0127 | - | >0.9999 | >0.9999 |
| **Left Paw** | >0.9999 | 0.2575 | 0.0127 | >0.9999 | - | >0.9999 |
| **Right Paw** | >0.9999 | 0.2575 | 0.0127 | >0.9999 | >0.9999 | - |

**Table S9.** Statistical p-values obtained after performing a Kruskal Wallis and Dunn’s test as post hoc analysis of the number of cells per area at day one (orange) and day three (blue) after IV administration of DiR-labeled ECFCs.

|  | **Thorax** | **Abdomen** | **Left Limb** | **Right Limb** | **Left Paw** | **Right Paw** |
| --- | --- | --- | --- | --- | --- | --- |
| **Thorax** | - | 0.0080 | 0.1407 | >0.9999 | >0.9999 | 0.8920 |
| **Abdomen** | <0.9999 | - | >0.9999 | 0.0432 | 0.5178 | >0.9999 |
| **Left Limb** | <0.9999 | <0.9999 | - | 0.5178 | >0.9999 | >0.9999 |
| **Right Limb** | 0.6669 | <0.9999 | 0.0555 | - | >0.9999 | >0.9999 |
| **Left Paw** | 0.6669 | <0.9999 | 0.0555 | <0.9999 | - | >0.9999 |
| **Right Paw** | 0.6669 | <0.9999 | 0.0555 | <0.9999 | <0.9999 | - |

**Table S10.** Statistical p-values of the fluorescence emitted/area after *ex vivo* NIR imaging in mice administered IM (red) or IV (blue) with DiR-labeled ECFCs. Data were analyzed by Kruskal Wallis test and Dunn’s test.

|  | **Spleen** | **Kidneys** | **Lungs** | **Liver** | **Left limb** | **Right limb** | **Left Paw** | **Right Paw** |
| --- | --- | --- | --- | --- | --- | --- | --- | --- |
| **Spleen** | - | 0.7146 | >0.9999 | >0,9999 | 0.7146 | 0.7146 | 0.7146 | 0.7146 |
| **Kidneys** | >0.9999 | - | >0.9999 | 0.2181 | >0.9999 | >0.9999 | >0.9999 | >0.9999 |
| **Lungs** | >0.9999 | >0.9999 | - | >0.9999 | >0.9999 | >0.9999 | >0.9999 | >0.9999 |
| **Liver** | >0.9999 | >0.9999 | >0.9999 | - | 0.2181 | 0.2181 | 0.2181 | 0.2181 |
| **Left Limb** | 0.0008 | 0.0008 | 0.0008 | 0.0008 | - | >0.9999 | >0.9999 | >0.9999 |
| **Right Limb** | >0.9999 | >0.9999 | >0.9999 | >0.9999 | 0.0008 | - | >0.9999 | >0.9999 |
| **Left Paw** | >0.9999 | >0.9999 | >0.9999 | >0.9999 | 0.0008 | >0.9999 | - | >0.9999 |
| **Right Paw** | >0.9999 | >0.9999 | >0.9999 | >0.9999 | 0.0008 | >0.9999 | >0.9999 | - |

**Table S11.** Statistical analysis of the number of cells estimated per area after *ex vivo* NIR imaging in mice injected via IM (red) or IV (blue) with DiR-labeled ECFCs. P-value obtained in each organ comparison is showed. Data were analyzed by Kruskal Wallis test and Dunn’s post hoc test.

|  | **Spleen** | **Kidneys** | **Lungs** | **Liver** | **Left limb** | **Right limb** | **Left Paw** | **Right Paw** | |
| --- | --- | --- | --- | --- | --- | --- | --- | --- | --- |
| **Spleen** | - | 0.7146 | >0.9999 | >0.9999 | 0.7146 | 0.7146 | 0.7146 | 0.7146 |  |
| **Kidneys** | >0.9999 | - | >0.9999 | 0.2181 | >0.9999 | >0.9999 | >0.9999 | >0.9999 |  |
| **Lungs** | >0.9999 | >0.9999 | - | >0.9999 | >0.9999 | >0.9999 | >0.9999 | >0.9999 |  |
| **Liver** | >0.9999 | >0.9999 | >0.9999 | - | 0.2181 | 0.2181 | 0.2181 | 0.2181 |  |
| **Left Limb** | 0.0008 | 0.0008 | 0.0008 | 0.0008 | - | >0.9999 | >0.9999 | >0.9999 |  |
| **Right Limb** | >0.9999 | >0.9999 | >0.9999 | >0.9999 | 0.0008 | - | >0.9999 | >0.9999 |  |
| **Left Paw** | >0.9999 | >0.9999 | >0.9999 | >0.9999 | 0.0008 | >0.9999 | - | >0.9999 |  |
| **Right Paw** | >0.9999 | >0.9999 | >0.9999 | >0.9999 | 0.0008 | >0.9999 | >0.9999 | - |  |

**Table S12.** Statistical p-values calculated after amplification of human-specific Alu sequences of DNA extracted from indicated organs. Balb-c nude received DiR-labeled ECFCs either IM (red) or IV (blue), and sacrificed three days after injection. Data were analyzed by Kruskal Wallis test and Dunn’s post hoc test.

|  | **Spleen** | **Kidneys** | **Lungs** | **Liver** | **Left back muscle** |
| --- | --- | --- | --- | --- | --- |
| **Spleen** | - | 0.1550 | 0.0491 | 0.0491 | >0.9999 |
| **Kidneys** | >0.9999 | - | >0.9999 | >0.9999 | 0.2205 |
| **Lungs** | >0.9999 | >0.9999 | - | >0.9999 | 0.0732 |
| **Liver** | >0.9999 | 0.6698 | >0.9999 | - | 0.0732 |
| **Left back muscle** | 0.1230 | 0.0040 | 0.4390 | 0.8732 | - |
